# Atlanta Academy of Medicine

**Published:** 1874-01

**Authors:** James B. Baird

**Affiliations:** Reporter


### Atlanta Academy of Medicine.

JAMES B. BAIRD, M.D., Reporter.

October 27, 1873.

Dr. J. P. Logan in the chair.

Dr. W. F. Westmoreland reported the following case: Female, seventeen years of age, unmarried; well grown; physique moderately good. Both parents died of consumption. Menstruation regular—no pain. Eighteen months ago the right mammary gland began to feel painful, and since then it has gradually increased in size, so that it is now one-third or one-fourth larger than the left. For the last six months she has had the peculiar lancinating pains, which are supposed to be pathognomonic of malignant disease. On examination no distinct tumor can be felt, and it is not tender, still the gland is harder than natural, and there is acute pain on pressure. What is this disease?

Is it malignant? Some of the characters of malignant disease are present, while others are absent.

Has always considered malignant disease as commencing with a hard lump or tumor, and acutely sensitive. Not able to-day to detect that peculiar condition, and the sensitiveness which have always been regarded by him as pathognomonic.

The idea presented itself, whether it was not a neurosis connected with the uterus. She says—and also those attending her—that there is no menstral trouble. Would be glad to hear any suggestions which would throw light on the case.

Dr. Rauschenberg remarked that he was not prepared to give a clinical name to the disease. Does not agree with Dr. W. that enlargment of the gland and the lancinating pain are pathognomonic of malignant disease. Can't have enlargment of a part without increased nutrition, and may account for these pains as result of the irritation of the vaso-motor nerves reflected upon the sensory nerves.

Dr. Baird asked if both breasts would not be affected if it were connected with uterine disease?

Dr. W. F. Westmoreland thinks not necessarily. Has seen a number of cases connected with uterine disease in which one breast only would increase in size at every menstrual period.

Dr. Rauschenberg asked why the enlargment might not be a simple hypertrophy. Does not consider the tearing, lancinating pains as pathognomonic of malignancy. We have lancinating pains in liver and other organs when there is no malignant disease present.

Dr. J. G. Westmoreland thinks the condition is not due to any derangement of the uterine functions. When you have these severe lancinating pains, even though the uterus be affected, would not think there was any connection between them.

The lancinating pains he considers pathognomonic of malignant disease.

Dr. Logan, from the history of the case, and the fact that both parents died of tuberculosis, considers it a case of scrofulosis. Recent investigations have shown a very close relationship between these two diseases, and he thinks it is very evident in a large majority of cases. Has never seen a case of malignant disease in which there was no tumor present.

Dr. J. G. Westmoreland thinks it unusual for scrofulosis to pursue the course of this case.

Dr. Baird asked Dr. W. F. W. if he considered cancer as simply local, followed by constitutional symptoms, or as the local expression of a constitutional disease?

Dr. W. F. W. replied that this question had been under discussion for the last century. Some contend that it is local, and the constitutional symptoms are secondary, others that it is constitutional, and the tumor a local symptom of the disease. He is of the opinion that it may be either; from local irritation may have malignant disease as result. Take a case of phymosis; in a number of such cases you have malignant disease of penis. Never saw malignant disease of the penis except when there was a long prepuce or from phymosis. Favors an early operation, whether the disease is local or constitutional. Never saw a case which could be traced to hereditary taint.

Dr. Logan is strongly impressed with the idea that this is a scrofulous affection, and not malignant. Rarely see malignant disease in children of tuberculous parents, whereas you often meet with scrofulosis in children whose parents have died of consumption. Many of the symptoms of malignancy are absent in this case. There is a strong tendency of opinion to a localization of the disease, and a very decided sentiment in favor of an early operation. Dr. Woodward, of Washington, has been investigating this subject, and read a very interesting paper favoring this view at the last meeting of the American Medical Association. Saw a report recently in a medical journal of a number of cases that had been operated upon, and after a number of years there were no symptoms of a return of the disease.

Dr. Baird reported a case of herpes zoster. Saw the case about ten days ago. Patient, female, about fourteen years of age, somewhat anæmic. Remarked that it was a beautifully developed case, though nothing very striking about it. The vesicles were as large as the end of the finger, and extended in a band four inches wide along the course of the ribs from spinal column to the mesial line in front.

Treatment—Tinc. Muriate Iron, min. 15, three times a day, and powdered Ox. Zinc locally. Has seen it stated that herpes zoster is always preceded by intercostal neuralgia; there was none in this case.

---

November 10th, 1873.

Dr. Jno. G. Westmoreland, First Vice-President, presiding.

Dr. W. F. Westmoreland stated that he had made a more careful examination of the case of suspected disease of the mammary gland, reported by him at the last meeting. He discovered that the pain was not confined to the mammary gland or to one side, but extended to the opposite side. Pressure applied over the sixth or eighth dorsal vertebra invariably produced the same pain. Four or five hours after this examination, was sent for and found his patient suffering intensely, the pain extending even to the legs.

He applied a slight Faradaic current, one pole being held in hand of the patient, the other placed lightly over the skin in the region of the sixth or seventh dorsal vertebra. In less than three minutes entire relief from pain was enjoyed, and there was no return of the violent paroxysms for a week, though there was slight daily pain, and the current is used daily. At the end of a week there was another paroxysm; since that time she has been entirely free from pain. The ulcer has healed. The breast has assumed almost its normal size. Thinks it a neurosis affecting intercostal nerves. Ten or twelve days had elapsed since the first application of the electricity.

Dr. Battey had met with a case in a lady, approaching the climacteric period, presenting the same symptoms, though the neuralgic pain was not so severe. There existed a tumor in one breast; shooting pains in breast and arm. No retraction of the nipple, and no fluctuation. He made an exploratory incision into the breast and evacuated a strumous abscess. Immediate relief was obtained. Adjourned.

---

NOVEMBER 17TH, 1874.

Dr. Ch. Rauschenberg, Third Vice-President, presiding.

Dr. Jno. G. Westmoreland called attention to a fever that had prevailed in this city during the fall and early winter, resembling very closely typhoid fever. The duration is longer than in ordinary cases of uncomplicated typhoid fever. All of his cases have continued for five weeks; generally typhoid fever lasts only three weeks.

Dr. G. W. Holmes: Was called to see a lady several weeks ago, supposed to be suffering from hæmorrhoids. She stated that she suffered pain resembling uterine pains during parturation. Reported that her bowels were kept regular by the habitual use of cold water enemata. Upon a digital examination he found a hard substance filling the rectum, with a perforation admitting the finger, which suggested the idea of a cylinder of wood. Diagnosis: Impacted fæces perforated through the center by the pipe of the syringe, through which perforation the alvine discharges took place. This hardened mass was gradually broken down by the finger, until the hand could be introduced into the rectum. He succeeded in this way in removing all—about one quart of hard, dry fæcal matter. Warm water and olive oil were then introduced, and the patient put upon a tonic treatment of quinine, strychnia and aloes. Recovery was immediate and complete.

Dr. W. F. Westmoreland reported the case of a gentleman in whom it was necessary to remove hard and impacted fæces from the rectum with the finger.

Dr. Baird: Called attention to the value of a solution of ox gall ʒj, to water Oss, injected into the rectum and retained for half an hour as a solvent for hardened fæces.

Dr. Love: Had a patient who suffered from obstinate constipation. Was in the habit of using injections. Frequently suffered more during a fæcal evacuation than in parturition. She ultimately had fissure of the anus, which was cured by a surgical operation. She was in the habit of going one and two weeks, and on one occasion, eighteen days without an action on her bowels. He was surprised at the extremely flaccid and dilated condition of the rectum. Strychnia and belladonna topically and subsequently, in minute doses, *per orem*, with cold injections into the rectum afforded relief. Gives belladonna to stimulate the nerves, through its influence on the ganglionic system. Regards it as a remedy *par excellence* in constipation.

Dr. Rauschenberg thought the rectum of women was capable of greater distention than in men, and that a greater pressure is required to produce a desire for evacuating the bowel, this, together with neglect in regard to a regular evacuation, makes constipation more common in females than in males. Thought that perseverance in regular attention to this duty would overcome it in any case.

Drs. W. F. Westmoreland, Holmes and Love, all agreed with Dr. R., in reference to the efficacy of regular attention to the act of defacation in overcoming habitual constipation.

Dr. Holmes reported a case of hæmorrhoids relieved by habitually evacuating the bowels just before retiring for the night. The cure was due to an empty rectum, and at the same time the horizontal position.

Dr. W. F. Westmoreland reported a case of gonorrhœal ophthalmia in an infant two or three days old, and asked for an expression of opinion as to the best mode of treatment.

Dr. A. W. Calhoun replied that in the majority of cases, by proper treatment, it could be prevented, as follows: When it is known that gonorrhœa exists, the vagina and labia should be kept thoroughly clean by frequent injections of cold water, or a weak solution of zinc, for several days before the birth of the child, and this should be continued up to the very moment of delivery. If this precaution is not taken, and ophthalmia neonatorum appears, the only treatment now adopted is the application, after everting the lids, of a strong solution of nitrate of silver—gr. x to ℥j, which may be neutralized by a solution of salt, according to the age of the patient and the discretion of the operator. One or two applications should be made a day, care being observed to carefully remove all of the secretions before applying the solution. If the scurf formed by the silver is too thick it can be neutralized by a solution of the chloride of sodium. To be efficient, the treatment should be adopted early, and the lids should be everted, otherwise the medicated fluid will not reach the palpebral portion of the conjunctival membrane. If the cornea becomes inflamed and ulcerated, perforation some times follows with prolapse of the iris, and advance and adhesion of the lens. If prolapse is large, clip off, and it is frequently necessary to touch with a little caustic. Little calcareous spots are frequently observed on the lens of children, situated just over the center of the pupil, and called central cataract. These opaque spots are due to perforation or ulceration of the cornea during intra-uterine life, or from the presence of a flake of lymph that has fallen on the lens from an inflamed cornea. There is no appearance of a cicatrix, except as is shown by the microscope in certain cases after death. Yellow oxide of mercury sometimes excites absorption of opacities of the cornea, but the most popular method of treatment, when the opacity is situated over the pupil, is by "tattooing" the cornea with India ink. This operation removes the deformity but does not restore the sight. It is done as follows: The lids are fixed and the opaque spot is repeatedly pierced by a number of fine needles fixed in a staff or handle. Dry India ink is spread over this, and the needles again applied; this is continued for several minutes, and the process should be repeated as often as necessary, generally three or four times, at intervals of one week. Usually, no inflammation is set up. The cure is obviously effected by the simple introduction of a coloring matter, which is not absorbed, into the spot on the cornea.

Adjourned.

---

NOVEMBER 24, 1873.

Dr. John G. Westmoreland, First Vice-President, presiding.

Dr. A. W. Calhoun reported the following case: A lady, æt. seventy-two. Three weeks ago operated on her for cataract. Used no anæsthetic, and gave no opiate. Nothing unusual occurred during the operation. The extraction was entirely successful. Immediately after the removal of the lens nausea and vomiting and considerable mental excitement occurred; the eye was dressed rapidly, and efforts made to soothe her. Vomiting continued occasionally. Saw her three hours after, and found her in a comatose state, with stertorous breathing, presenting the symptoms of apoplexy. She died half an hour later. Thinks apoplexy was unquestionably the cause of death. Was the rupture of the vessel—assuming this to have occurred—due to mental excitement or efforts at vomiting, or was the nausea and vomiting the effect of the effusion?

Dr. Battey inquired if there was much loss of vitreous.

Dr. Calhoun replied that there was no loss of vitreous humor, and added that the vision of the patient was as perfect as ever under similar circumstances; she recognized fingers at the distance of two feet, the usual test; and also recognized the faces of friends not seen for four and five years.

Dr. Battey inquired as to Dr. C.'s observation in respect to nausea after cataract operations?

Dr. Calhoun replied that it does not often occur, and does not depend upon loss of vitreous, unless as much as one-half of the vitreous is lost, then nausea is produced. He has seen hundreds of operations for cataract, and only two or three, and those delicate persons, affected with nausea, unless produced by the administration of an anæsthetic or an anodyne. Referred to the case of an old gentleman he had seen operated on in Germany for cataract. He soon became comatose, and his life was despaired of; though he rallied, and ultimately recovered.

Dr. Baird asked what relation existed between the loss of vitreous and nausea.

Dr. Calhoun announced that it had never been satisfactorily explained, and requested the opinion of the members of the Academy.

Dr. Battey called attention, as a step towards an explanation, to experiments made some years ago in France, in which a species of catalepsy was produced by requiring the subject to gaze intently upon a bright silver ball, suspended by a band fastened around the forehead. Once saw a large mammary abscess opened by an extensive incision, when the patient was in this cataleptic state, and she did not give the slightest evidence of pain.

Dr. Love cited, as examples of mental impressions made through the medium of the nerves, the vertigo occasioned by walking over a high tressel, or on a log over a stream of water. He had also known of vomiting produced by reflex action, when the retina was exposed alternately to light and shade, as walking near a picket fence, through which the sun was shining.

Dr. Battey stated that his own personal experience confirmed the views expressed by Dr. Love.

Dr. Holmes thought, in reference to the case reported by Dr. Calhoun, that the nausea was caused by mental excitement, and that the vessels of the brain being softened, as is common in persons in advanced age, were ruptured by a slight determination of blood to the brain, the result of the excitement, and the efforts at vomiting.

Dr. Love reported the case of a lady, æt. thirty, married, and the mother of one child. First seen six weeks ago. She was then suffering from nausea, occasional vomiting, and syncope; no fever; pulse weak and soft; could detect no lesion; condition of skin good; mind clear; bowels regular; digestion good; urine normal; uterus healthy. She had been forsaken by her husband, and reference to this subject invariably produced a paroxysm of vomiting and fainting. He gave stimulants and anti-spasmodics, and ordered a nutritious diet. Two weeks after she had excessive hemorrhages from the bowels. Temperature slightly raised, though never above 101° F. Five days later she died, seemingly from simple exhaustion. He regarded her condition as due alone to mental disturbance, and asked for opinions in regard to the influence of excitement and mental emotions in hemorrhages and vomiting.

Dr. Battey said that some years ago he had a case in a strong, robust, though intemperate man, who suddenly passed a large quantity of blood, perhaps a half gallon, from his bowels, and ejected half as much from his stomach. In a week he was well, on the streets. Thinks it came from ruptured capillaries of the intestines and stomach.

Dr. Holmes asked if it was possible for blood to pass through the tissues—to transude. He gave a negative opinion.

Dr. W. F. Westmoreland stated that if blood in any quantity is poured out, it proves that a vessel has been ruptured. Regarded Dr. Love's case as typhoid fever.

Dr. Love remarked that Kolliker and Virchow say that blood globules may pass through tissues. Though, when it flows in large quantities, it must come from ruptured vessels. Thinks the temperature in his case not high enough for typhoid fever.

Dr. W. F. Westmoreland thought the thermometer utterly worthless in diagnosis and prognosis. The idea that typhoid fever must have one temperature, and pneumonia and other diseases another temperature is absurd. Has seen typhoid fever in which the patient did not keep his bed.

Adjourned.

